# Molecular subtyping of metastatic melanoma based on cell ganglioside metabolism profiles

**DOI:** 10.1186/1471-2407-14-560

**Published:** 2014-08-01

**Authors:** Cristina Tringali, Ilaria Silvestri, Francesca Testa, Paola Baldassari, Luigi Anastasia, Roberta Mortarini, Andrea Anichini, Alejandro López-Requena, Guido Tettamanti, Bruno Venerando

**Affiliations:** Department of Medical Biotechnology and Translational Medicine, University of Milan, Segrate, Milan Italy; Human Tumors Immunobiology Unit, Department of Experimental Oncology and Molecular Medicine, Fondazione IRCCS Istituto Nazionale dei Tumori, Milan, Italy; Laboratory of Stem Cells for Tissue Engineering, IRCCS Policlinico San Donato, San Donato Milanese, Milan, Italy; Department of Biomedical Sciences for Health, University of Milan, Segrate, Milan Italy; Immunobiology Department, Center of Molecular Immunology, Havana, Cuba

**Keywords:** Ganglioside, Melanoma, N glycolyl GM3, Sialidase, Survival

## Abstract

**Background:**

In addition to alterations concerning the expression of oncogenes and onco-suppressors, melanoma is characterized by the presence of distinctive gangliosides (sialic acid carrying glycosphingolipids). Gangliosides strongly control cell surface dynamics and signaling; therefore, it could be assumed that these alterations are linked to modifications of cell behavior acquired by the tumor. On these bases, this work investigated the correlations between melanoma cell ganglioside metabolism profiles and the biological features of the tumor and the survival of patients.

**Methods:**

Melanoma cell lines were established from surgical specimens of AJCC stage III and IV melanoma patients. Sphingolipid analysis was carried out on melanoma cell lines and melanocytes through cell metabolic labeling employing [3-^3^H]sphingosine and by FACS. N-glycolyl GM3 was identified employing the 14 F7 antibody. Gene expression was assayed by Real Time PCR. Cell invasiveness was assayed through a Matrigel invasion assay; cell proliferation was determined through the soft agar assay, MTT, and [^3^H] thymidine incorporation. Statistical analysis was performed using XLSTAT software for melanoma hierarchical clustering based on ganglioside profile, the Kaplan-Meier method, the log-rank (Mantel-Cox) test, and the Mantel-Haenszel test for survival analysis.

**Results:**

Based on the ganglioside profiles, through a hierarchical clustering, we classified melanoma cells isolated from patients into three clusters: 1) cluster 1, characterized by high content of GM3, mainly in the form of N-glycolyl GM3, and GD3; 2) cluster 2, characterized by the appearance of complex gangliosides and by a low content of GM3; 3) cluster 3, which showed an intermediate phenotype between cluster 1 and cluster 3. Moreover, our data demonstrated that: a) a correlation could be traced between patients’ survival and clusters based on ganglioside profiles, with cluster 1 showing the worst survival; b) the expression of several enzymes (sialidase NEU3, GM2 and GM1 synthases) involved in ganglioside metabolism was associated with patients’ survival; c) melanoma clusters showed different malignant features such as growth in soft agar, invasiveness, expression of anti-apoptotic proteins.

**Conclusions:**

Ganglioside profile and metabolism is strictly interconnected with melanoma aggressiveness. Therefore, the profiling of melanoma gangliosides and enzymes involved in their metabolism could represent a useful prognostic and diagnostic tool.

**Electronic supplementary material:**

The online version of this article (doi:10.1186/1471-2407-14-560) contains supplementary material, which is available to authorized users.

## Background

Melanoma is the most lethal form of skin cancer and accounts for at least 48,000 deaths worldwide annually [1, 2]. Clinical outcome of melanoma largely depends on the tumor stage upon first diagnosis. Currently, melanoma staging is based on the guidelines published by the American Joint Committee on Cancer (AJCC) in 2009 [3] and advises the employment of histopathological and clinical criteria. Nevertheless, this system is limited in its ability to provide a precise prognosis: a large number of patients with similar or identical clinical and histopathological features has different clinical outcome, from being cured to death [4]. Also, the identification of some mutations concerning oncogenes, including *BRAF*, *NRAS*, *KIT*, has been shown to be very useful to predict the response to therapy, but it is not clear whether the determination of the mutational status could offer any prognostic value [5]. Many attempts have been done to identify molecular markers or gene and protein signatures predicting clinical outcome in melanoma but, so far, none of them has been sufficiently validated [6–9].

Among the distinctive molecular markers displayed by melanoma cells, many studies identified specific gangliosides not detectable in normal melanocytes [10]. A pivotal role played by gangliosides was recognized in the development of melanoma and in its biological features [11]. In particular, GD3 and derivatives [12, 13], which are also present at high levels in proliferating cells [14–16], have been demonstrated to promote melanoma cell proliferation and invasion [17]. Moreover, de-N-acetyl GM3 (d-GM3) (a variant of GM3 with a free amino group at the 5 position of sialic acid instead of the acetyl group) was found in melanoma and it was demonstrated that it enhanced cell migration and invasion [18]. Also, N glycolyl GM3 (Neu5Gc-GM3) (a variant of GM3 that contains N-glycolylneuraminic acid instead of N-acetylneuraminic acid) was recognized in melanoma, even its role in this disease is still obscure [19]. The alteration of the expression/catalytic activity of glycosyltransferases and enzymes involved in gangliosides metabolism could be critical factors in determining the melanoma cell ganglioside composition. In particular, the activation of GD3 synthase is linked to the increment of GD3 in melanoma cells [11, 20]. Similarly, the plasma membrane sialidase *NEU3* has been shown to be highly expressed in human melanoma cell lines [21].

Prompted by these data, we sought to investigate the ganglioside metabolism profile of metastatic melanoma cell lines established from patients. Our results demonstrated that: a) melanomas displayed different ganglioside patterns and three clusters of tumors could be identified; b) a correlation could be traced between patients’ survival and melanoma ganglioside profiles; c) the expression of several enzymes involved in ganglioside metabolism was associated with patients’ survival; d) melanoma clusters identified on the basis of ganglioside profile exhibited different features determining melanoma malignancy.

## Methods

### Cell cultures

Melanoma cell lines were established from surgical specimens of AJCC stage III and IV melanoma patients admitted to Fondazione IRCCS Istituto Nazionale dei Tumori, Milan [22, 23]. Molecular and biological characterization of the cell lines has been reported previously [24]. All cell lines were maintained as described [25]. All patients were informed about the scope and methods and delivered a written informed consent for the use of the surgical samples to establish cell lines. The study was approved by the Ethics Committee of the University of Milan and was performed according to the Declaration of Helsinki.

Clones 2/14 and 2/21 were isolated from a single human metastatic melanoma cell line, as described [26, 27]. NHEM-Ad and NHEM-Neo were purchased by Lonza (Basel, Switzerland) and PromoCell (Heidelberg, Germany), and maintained in mMGM-4 medium (Lonza).

### Sphingolipid analysis

Sphingolipid analysis was carried out through cell metabolic labeling with [3-^3^H]sphingosine (PerkinElmer, Waltham, MA, USA) [28]. In order to assay the hypothesis that Neu5Gc-glicans could be incorporated from the culture medium and then employed for the synthesis of GM3, before [3-^3^H]sphingosine labeling, melanoma L6 cells were pre-incubated in the reduced-serum medium OptiMEM (Life Technology, Carlsbad, CA, USA) for 5 days.

Ganglioside and neutral sphingolipid extracts were analyzed by HPTLC carried out with the solvent systems chloroform/methanol/0.2% CaCl_2_ 55:45:6 (v/v) and chloroform/methanol/water 110:40:6 (v/v), respectively. To separate Neu5Gc-GM3 from Neu5Ac-GM3, HPTLC was carried out using the solvent system chloroform/methanol/0.2% CaCl_2_/5 N NH_3_ 50:42:6:4 (v/v). The sphingolipid pattern was determined and quantified by radiochromatoscanning (Betaimager 2000, Biospace, Paris, France) [28, 29].

Endogenous sphingolipid analysis performed to standardize metabolic labeling was performed as previously described [30].

Ganglioside standards were kindly given by Prof. Sonnino, University of Milan.

### Immunostaining of HPTLC

After the chromatographic separation of gangliosides, the plates were soaked in acetone plus 0.1% polyisobutylmethacrylate. After drying and blocking with PBS-4% milk, the plates were incubated with 5 μg/ml of anti-Neu5Gc-GM3 murine 14 F7 antibody [31, 32], overnight, and, then, with a horseradish peroxidase-conjugated anti-mouse IgG antibody (Santa Cruz Biotechnology, Santa Cruz, CA, USA). The reaction was stained with the SuperSignal West Pico Chemiluminescent Substrate (Thermo Fisher Scientific, Dallas, MA, USA).

### Immunohistochemical analysis

Cytospin preparations of melanoma cell lines were fixed with formalin for 10 min at room temperature and then incubated with 14 F7 mAb, as described [19]. The slides were then incubated with goat anti-mouse biotinylated secondary antibody, followed by streptavidin/HPR (Dako, Glostrup, Denmark), for 30 min at each step. The enzyme activity was detected with a commercial solution of 3, 3′-Diaminobenzidine (Sigma-Aldrich, St Louis, MO, USA). Cells were counterstained with Mayer’s Hematoxylin (Bio-Optica, Milan, Italy). Slides subjected to the same treatments, but without incubation with primary mAb, were used as negative controls. Images were acquired at × 20 with an Axiovert 100 microscope (Zeiss, Carl Zeiss, Thornwood, NY) equipped with a digital camera (AxioCam MrC5, Zeiss).

### Phenotypic profile of melanoma cell lines

Cell surface expression of GM3, GD2 and GD3 was determined in representative melanoma cell lines belonging to different clusters, by staining with the following mAbs: anti GM3 (Neu5Ac-GM3) M2590 (Cosmo Bio Co, LTD, Tokyo, Japan), anti-human disialoganglioside GD2 (BD Pharmingen, San Diego, CA, USA), anti-ganglioside GD3 (R24) (Abcam Inc., Cambridge, UK), followed by a FITC-conjugated F(ab’)_2_ fragment goat anti-mouse Ab (Jackson ImmunoResearch, West Grove, PA) as described [33]. Samples were acquired by a fluorescence-activated cell sorting (FACS)-Calibur cytofluorimeter (BD Biosciences) and analyzed by the FlowJo software (Tree Star, Ashland, OR, USA). Results were expressed as percentage of positive cells and as mean fluorescence intensity after subtraction of mean fluorescence intensity in cells stained with secondary antibody only.

In order to compare the phenotype of different tumors, all main FACScan operative settings were kept constant throughout the whole study.

### PCR and Real-time RT-PCR

*CMAH* expression was assayed in cDNA isolated from melanocytes and melanoma cells through RT-PCR. The genomic region corresponding to the 92-bp deletion in the human *CMAH* gene was amplified by PCR using genomic DNA extracted from melanoma cells and from murine C2C12 cells, as positive control. Real Time PCR was performed as previously reported [34]. Primer sequences are reported in Table 1.

**Table 1:** **Primers used for gene expression**

Gene	Forward primer	Reverse primer
GD3 synthase (*ST8SIAI*)	5′-CAGCATAATTCGGCAAAGGT-3′	5′-ATTGGCACCAACATCTGACA-3′
*NEU1*	5′-CCTGGATATTGGCACTGAA-3′	5′-CATCGCTGAGGAGACAGAAG-3′
*NEU2*	5′-AGAAGGATGAGCACGCAGA-3′	5′-GGATGGCAATGAAGAAGAGG-3′
*NEU3*	5′-GGCTTGTTTGGGTGTTTGTT-3′	5′-CATCGCTGAGGAGACAGAAG-3′
*NEU4*	5′-ACCGCCGAGAGTGTTTTGG-3′	5′-CGTGGTCATCGCTGTAGAAGG-3′
GM3 synthase (*ST3GAL5*)	5′-CCCTGAACCAGTTCGATGTT-3′	5′-CATTGCTTGAAGCCAGTTGA-3′
GM1 synthase (*B3GALT4*)	5′-CGCCTTCCAGGACTCCTACC-3′	5′-CCGTCTTGAGGACGTATCGG-3′
GM2 synthase (*B4GALNT1*)	5′-TCTCACTGGAGAGGGTCAGG-3′	5′-CGGACTGTGTCTGCTGTGTT-3′
GD1a synthase (*ST6GALNAC4*)	5′-TCTACCACCCAGCCTTCATC-3′	5′-TAGTGGTGCCAGTTCCCTTT-3′
*ST3GalVI*	5′-TTGCCCTATGGGATGAGAAC-3′	5′-CCTCCATTACCAACCACCAC-3′
*ST3GalNac6*	5′-CGCCGGAGAGAAATGAGTA-3′	5′-CCACTTCTTGAGGTTGACAGG-3′
*SP1*	5′-GGCTACCCCTACCTCAAAGG-3′	5′-CACAACATACTGCCCACCAG-3′
*CMAH*	5′-GAATTTATTGTCCCTTTGC-3′	5′-TATGATGCCCTTGCTGTC-3′
*CMAH* deletion	5′-AGCTTTCCGGAGGGTTGATA-3′	5′-TGTCAGATGCACAAGCACAA-3′
*ACTB* (β actin)	5′-CGACAGGATGCAGAAGGAG-3′	5′-ACATCTGCTGGAAGGTGGA-3′

### Invasiveness assay

Melanoma cell invasiveness was assayed through a Matrigel assay, as previously reported [35].

### Proliferation assays

Soft agar, thymidine incorporation, and MTT assays were performed as previously reported [34].

### Sialidase activity toward endogenous cell gangliosides

To test the ability of melanoma plasma membrane sialidase to act on endogenous cell gangliosides, melanoma cell particulate fractions, obtained as previously described [36], were incubated with [3-^3^H]sphingosine labeled cell gangliosides at pH 3.8, overnight, at 37°C. Then, gangliosides were fractionated by HPTLC and visualized by radiochromatoscanning.

### Statistical analysis

Hierarchical clustering was done using XLSTAT software. Survival analysis was carried out by the Kaplan-Meier method; survival curves were compared by the log-rank (Mantel-Cox) test; hazard ratios were computed by the Mantel-Haenszel test. Ganglioside composition, Real Time PCR data, [^3^H]thymidine incorporation were compared using Student t-test and 1-way ANOVA.

## Results

### Melanoma cells can be clustered on the basis of their ganglioside profile

Sphingolipid pattern was determined in 23 short-term cell lines established from 5 primary VPG (vertical growth phase) melanomas and from 18 lymph nodes metastases as well as in adult (NHEM-Ad) and neonatal (NHEM-Neo) melanocytes. To this end, [3-^3^H]sphingosine was administrated to the cells leading to an extensive labeling of all sphingolipids [37]. The treatment was performed in order to reach a metabolic steady state that corresponded to endogenous non-radiolabelled cell sphingolipid pattern. NHEM-Ad and NHEM-Neo melanocytes ganglioside profile was mainly composed of GM3 and GD3 (in traces in NHEM-Ad cells and more abundant in NHEM-Neo cells) with traces of sialyl-paragloboside (SPG) (Additional file 1).

**Figure 1:**
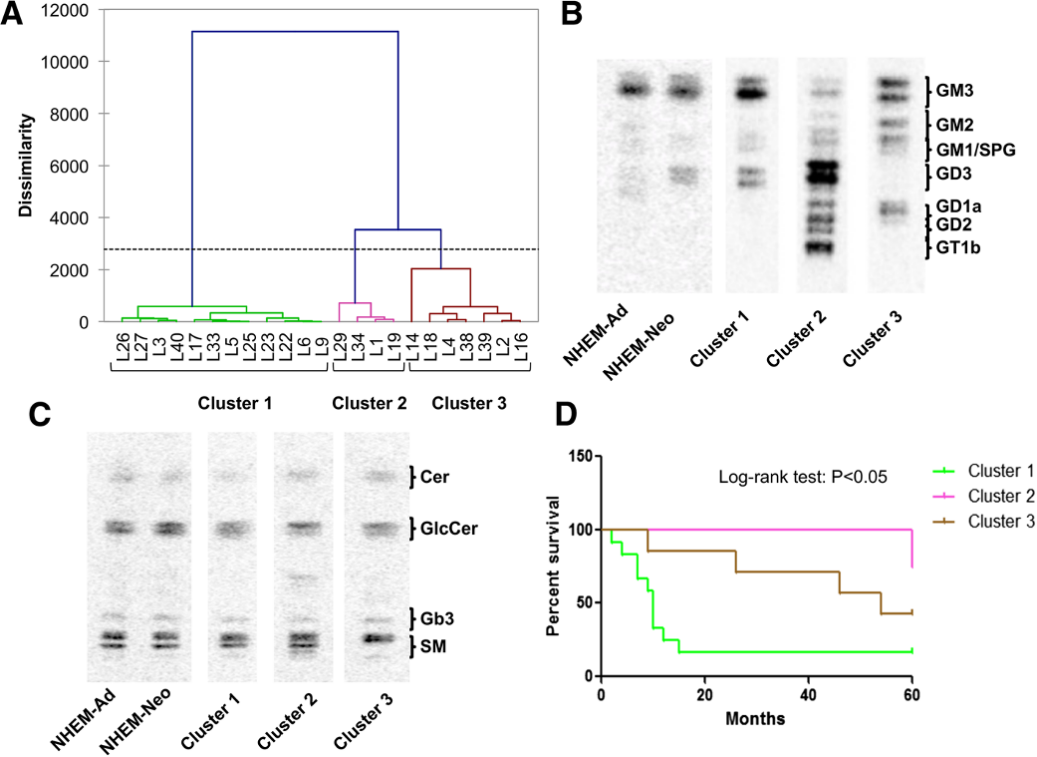
**Association of melanoma cell ganglioside profile with patients’ survival. (A)** Hierarchical clustering analysis of melanoma cell lines on the basis of ganglioside profiles. Dotted line represents the level of significant dissimilarity. **(B)** HPTLC separation of gangliosides and **(C)** of neutral sphingolipids of three representative melanoma cell lines (L6, L34, L4) belonging to clusters 1, 2, and 3, in comparison to adult and neonatal melanocytes. **(D)** Kaplan-Meier survival analysis of patients according to the three clusters identified. Survival was evaluated as time from first diagnosis.

The distinct ganglioside profiles of melanoma cells allowed us to classify them into three main groups (Figure 1A). The ganglioside HPTLC pattern of NHEM-Ad and NHEM-Neo melanocytes and of representative cell lines of each cluster is shown in Figure 1B. Melanoma cells belonging to cluster 1 showed a strong similarity with neonatal melanocytes, with GM3 as the main ganglioside and an increased amount of GD3 (Additional file 1, Figure 1B). Instead, ganglioside profiles of cells belonging to clusters 2 and 3 were significantly different from that of melanocytes, revealing the appearance of novel species such as GM2, GD1a, GD2, GT1b in cluster 2, and GM2, GD1a, GD2 in cluster 3, in addition to GM3 and GD3 (Additional file 1, Figure 1B). No significant differences were found considering primary lesions versus metastases or the clinical stage of the disease at the time when tumors were surgically removed and cell lines established (AJCC stage III versus stage IV) (Additional file 1).

Despite these huge differences regarding the ganglioside profiles of the three clusters, neutral sphingolipid pattern was quite similar also to adult and neonatal melanocytes pattern, except for a 30-40% decrease of ceramide amount in cells classified in cluster 1 in comparison to cells classified in cluster 2 (P < 0.05) (Figure 1C).

Kaplan-Meier survival analysis and log-rank test demonstrated that patients whose tumors fell in cluster 1 had a significantly worse overall survival, measured from the time of the first diagnosis, in comparison to patients whose tumors were classified in cluster 3 and, above all, in cluster 2 (P < 0.05) (Figure 1D). Patients belonging to cluster 1 had a hazard ratio of 4.23 in comparison to patients belonging to cluster 2 (P < 0.05), and a hazard ratio of 2.91 as compared with patients belonging to cluster 3 (P < 0.05).

**Figure 2:**
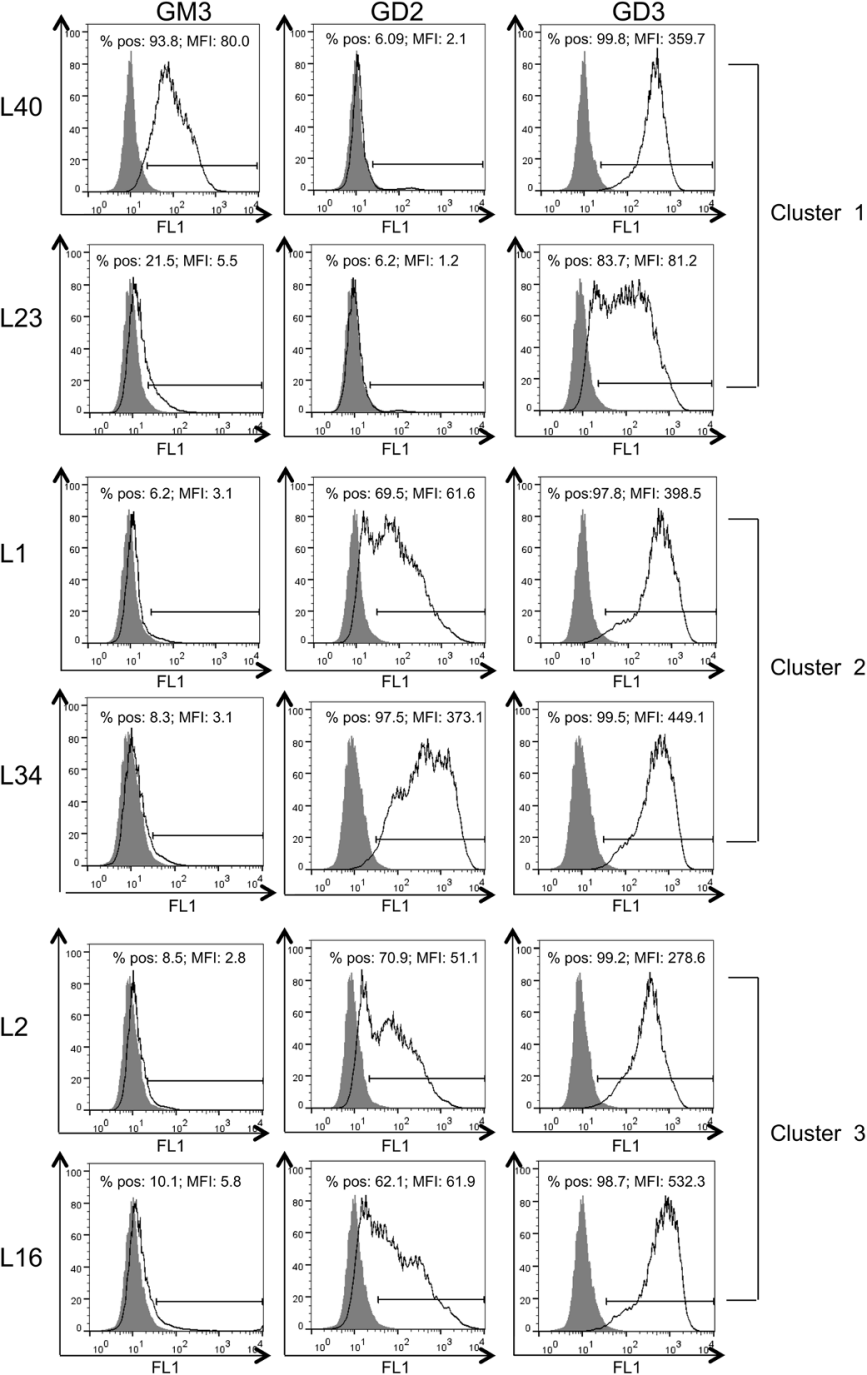
**Ganglioside phenotypic profile of melanoma cell lines.** Cell surface staining of GM3, GD2, and GD3 by mAbs: anti Neu5Ac-GM3 (M2590); anti-GD2, anti-GD3 (R24), determined in representative melanoma cell lines belonging to different clusters (L40 and L23 for cluster 1; L1 and L34 for cluster 2; L2 and L16 for cluster 3).

The ganglioside phenotypic profile of representative cell lines for each cluster is reported in Figure 2. A low heterogeneity was observed concerning the content of GD3 and GD2 in some cell lines.

### A high amount of N-glycolyl GM3 characterizes melanoma cluster 1 cells

**Figure 3:**
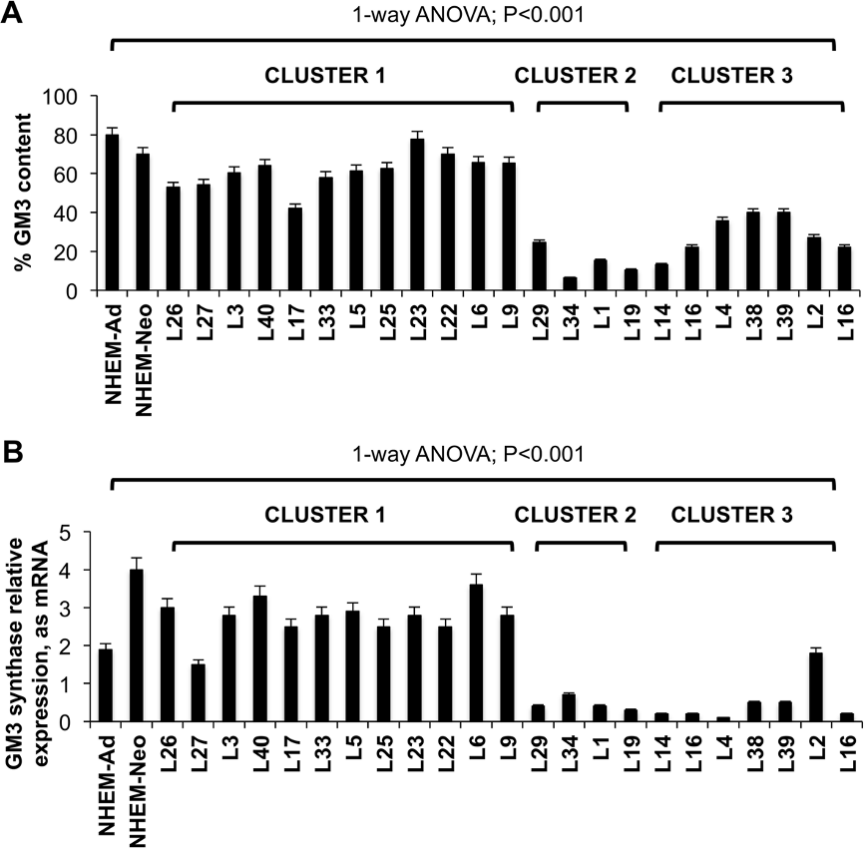
**Correlation between GM3 amount and melanoma cell malignancy. (A)** GM3 content and **(B)** Real Time PCR analysis of the mRNA expression of GM3 synthase in melanoma cells and in melanocytes. Values are the mean ± S.D. of four independent experiments.

**Figure 4:**
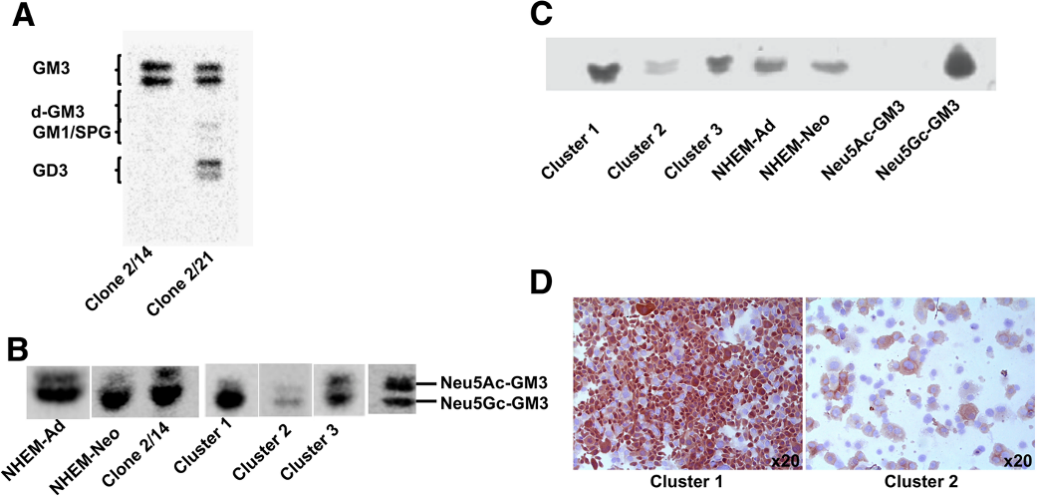
**Correlation between Neu5Gc-GM3 and melanoma cell malignancy. (A)** HPTLC separation of gangliosides of two cell clones (2/14 and 2/21) isolated from a single patient. **(B)** HPTLC separation of Neu5Ac-GM3 and Neu5Gc-GM3 in NHEM-Ad, NHEM-Neo, clone 2/14, and in melanoma cell lines representative of cluster 1 (L6), cluster 2 (L34), cluster 3 (L4). **(C)** 14 F7 antibody immunostaining of gangliosides separated by HPTLC (NHEM-Ad, NHEM-Neo, L6 representative of cluster 1, L34 representative of cluster 2, L4 representative of cluster 3. **(D)** Immunohistochemical analysis of cytospin preparations of L6 representative of cluster 1 and L34 representative of cluster 2, stained with 14 F7 antibody. Original magnification, x20 (Axiovert 100). Similar results were obtained for other melanoma cell lines according to the clustering.

We recorded a singular trend showing a marked decrease of the average GM3 per cent content from melanoma cluster 1 cells to cluster 3 (-58.3%; cluster 3 versus cluster 1, P < 0.001), and to clusters 2 (-76.5%; cluster 2 versus cluster 1, P < 0.001) (Figure 3A). This trend was associated with the decrease of GM3 synthase expression in clusters 2 and 3, in comparison to cluster 1 (-85.7%, cluster 2 versus cluster 1; -82.1% cluster 3 versus cluster 1; P < 0.001) (Figure 3B). To confirm the key role played by GM3 in metastatic melanoma behavior, we analyzed the ganglioside composition of two melanoma clones (2/14 and 2/21) isolated from a single melanoma cell line [26]. These two clones had markedly different molecular and biological features [26, 27], including adhesion molecule phenotype, degree of differentiation, recognition by antigen-specific T cells, presence of mutated NRAS (clone 2/14) or mutated BRAF (clone 2/21), growth in immunodeficient mice (enhanced in clone 2/14) [26, 27]. Significantly, the ganglioside profile of clone 2/14 consisted only of GM3 (Figure 4A).

**Figure 5:**
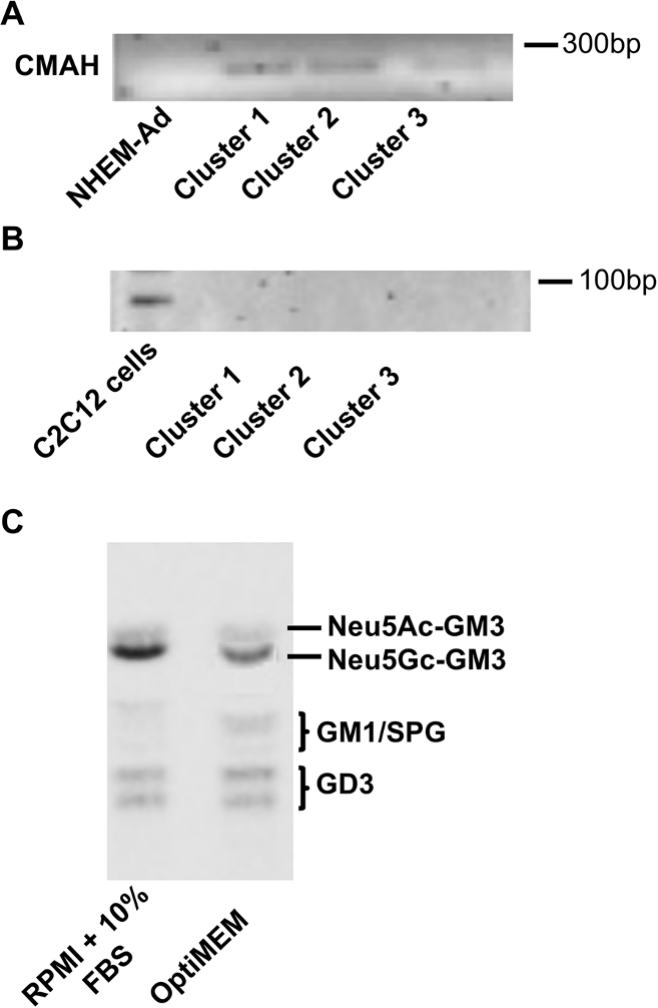
***CMAH*** **in melanoma cells. (A)** RT-PCR analysis of *CMAH* expression in L6 (cluster 1), L34 (cluster 2), L4 (cluster 3). **(B)** RT-PCR analysis of deletion at 5′ of *CMAH* gene in L6 (cluster 1), L34 (cluster 2), L4 (cluster 3). Similar results were obtained for other melanoma cell lines according to the clustering. **(C)** HPTLC separation of gangliosides of L6 cells after pre-incubation in medium containing 10% FBS or in serum-reduced medium OptiMEM. Three replicate experiments were performed.

We assessed that a high amount of GM3 was present in clone 2/14, as well as in melanoma cell lines, as Neu5Gc-GM3. Neu5Gc-GM3 was detected also in melanocytes. d-GM3 was not present (Figure 4A). The increase of GM3 in melanoma cluster 1 cells reflected an increased content of Neu5Gc-GM3 (90% of total cellular GM3) in comparison to other clusters (Figure 4B). The identification of Neu5Gc-GM3 was validated by the employment of the Neu5Gc-GM3-specific antibody 14 F7 [19] in HPTLC immunostaining (Figure 4C) and in immunohistochemical analysis, which confirmed its enhanced amount in cluster 1 melanoma cells (Figure 4D). Because it was reported that N-glycolylneuraminic acid synthesis could not occur in human cells due to a 92 bp sequence deletion in the *CMAH* gene that blocks the synthesis of a functional form of CMP-NeuAc hydroxylase (*CMAH*), the enzyme responsible for the conversion of CMP-N-acetylneuraminic acid into CMP-N-glycolylneuraminic acid [38, 39], firstly we explored if this genetic alteration was reverted in melanoma cells. We recorded *CMAH* expression as mRNA in all melanoma cell lines, in contrast to melanocytes where it was null (Figure 5A). Nevertheless, we demonstrated that the *CMAH* gene maintained the 92 bp sequence deletion typical of human cells [40], also in melanoma cells (Figure 5B); therefore, on the basis of previous evidences [41], this gene cannot be translated into an enzymatically active protein. Many reports demonstrated that some cancer cells become able to incorporate dietary CMP-N-acetylneuraminic acid at a faster rate from biological fluids and therefore, they incorporate it into gangliosides. Effectively, we demonstrated that cultivating melanoma cells in a serum-reduced medium for 5 days, the content of Neu5Gc-GM3 was reduced by 25% (P < 0.01) (Figure 5C). Therefore, we concluded that melanoma cells do not gain the capability to synthetize CMP-N-acetylneuraminic acid but they can acquire it from external molecules.

### Correlation between glycosyltransferases expression and patients’ survival

**Figure 6:**
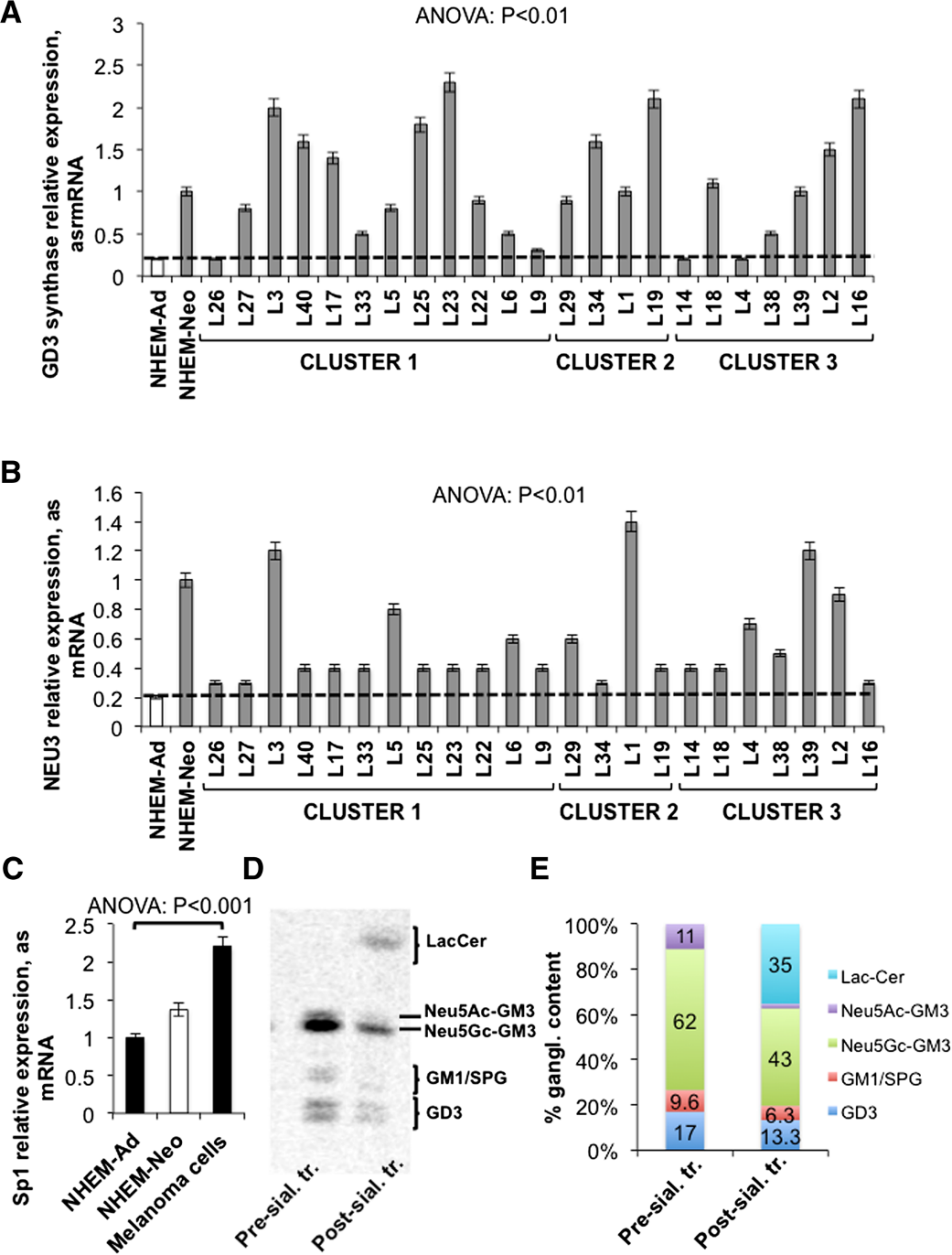
**Up-regulation of GD3 synthase and** ***NEU3*** **in melanoma cells. (A)** Real Time PCR analysis of the mRNA expression of GD3 synthase and **(B)** of *NEU3* sialidase in melanoma cell lines and in melanocytes. Dotted line represents GD3 synthase and *NEU3* expression level in adult melanocytes. Values are the mean ± S.D. of four independent experiments. **(C)** Average mRNA expression of *Sp1*, assessed by Real Time PCR, in melanoma cell lines and in melanocytes. Values are the mean ± S.D. of four independent experiments. **(D)** HPTLC separation and **(E)** percent quantification of melanoma cell (L6) gangliosides before and after incubation with melanoma cell membranes.

We confirmed an overall higher expression of GD3 synthase (11.5-1.5-fold) and plasma membrane sialidase *NEU3* (7–1.5-fold) in all melanoma cells than melanocytes (Figure 6A,B) [11, 21]. The increase of the expression of these two enzymes was reasonably induced by the enhanced expression of the transcriptional factors *Sp1* (average increase: 100%, melanoma cells versus adult melanocytes; P < 0.001) (Figure 6C), as it was reported that both *NEU3* [42] and GD3 synthase gene promoters [43] are regulated by them. *NEU*3 was the unique sialidase to be over-expressed by melanoma cells; lysosomal sialidase *NEU1* expression varied inconsistently among melanoma cell lines and melanocytes; cytosolic sialidase *NEU2* was not expressed in both melanocytes and melanoma cells; sialidase *NEU4* appeared to be down-regulated in all melanoma cells except L1, L34, L39, L40, in comparison to melanocytes. As melanoma cells expressed high levels of GM3, the increase of *NEU3* expression appeared a paradox. Therefore, we tried to assess if endogenous GM3 could be accessible to NEU3 activity in melanoma cells. To this end, after metabolic labelling with [3-^3^H]sphingosine, gangliosides were extracted from a representative melanoma cell line belonging to cluster 1 (L6) and incubated with membranes obtained by the same cell line, in order to perform an exhaustive treatment. We demonstrated that NEU3 was able to hydrolyze and convert into lactosylceramide only 41% of plasma membrane GM3: the aliquot of Neu5Gc-GM3 was particularly resistant to sialidase action (-25-30%), while N acetyl GM3 (Neu5Ac-GM3) was entirely hydrolyzed. Also, GD3 and SPG decreased by 22% and 34%, respectively, after incubation (pre-incubation gangliosides versus post-incubation gangliosides; P < 0.01) (Figure 6D,E).

**Figure 7:**
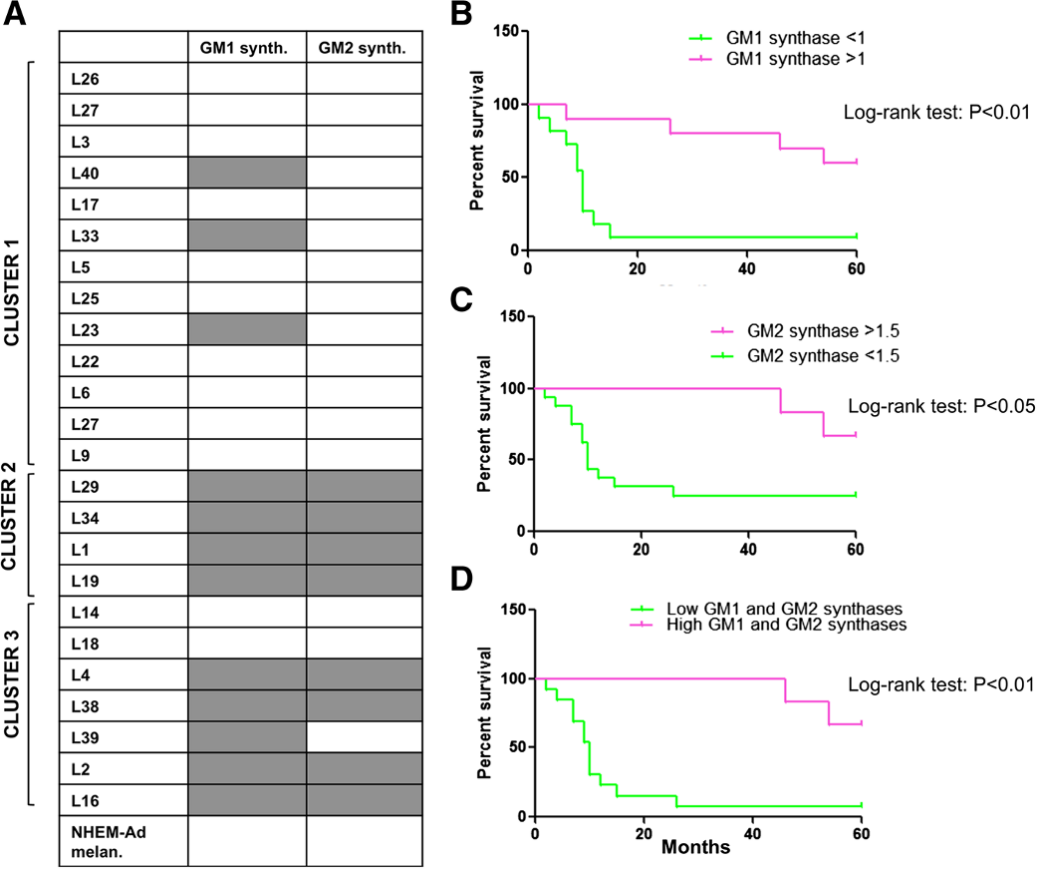
**Altered expression of GM1 and GM2 synthases in melanoma cells. Correlation with patients’ survival. (A)** GM1 and GM2 synthases expression assessed by Real Time PCR in melanoma cells separated in three clusters. Grey color denotes the expression of the enzyme, white color the absence. Values are the mean ± S.D. of four independent experiments. **(B)** Kaplan-Meier survival analysis of patients according to GM1 synthase expression, **(C)** according to GM2 synthase expression, and **(D)** according to both GM1 and GM2 synthases expression. Survival was evaluated as time from first diagnosis.

We recorded in some melanoma cell lines grouped, particularly, in clusters 2 and 3, the expression of GM1 and GM2 synthases that were not expressed by melanocytes (Figure 7A). The expression of GM1 and GM2 synthases was related to the appearance of GM1 and GM2 among the gangliosides of these cells (Additional file 1). Kaplan-Meier survival analysis and log-rank test demonstrated that patients whose tumors showed a relative mRNA expression of GM1 synthase, normalized on β actin, higher than 1 had a significantly better overall survival, measured from the time of the first diagnosis, in comparison to patients whose tumors showed a less relative mRNA expression of this enzyme (P < 0.01) (Figure 7B). Patients carrying tumors endowed with GM1 synthase low expression had a hazard ratio of 4.499 in comparison to other patients (P < 0.01). Similarly, patients whose tumors showed a relative mRNA expression of GM2 synthase, normalized on β actin, higher than 1.5 had a significantly better overall survival, measured from the time of the first diagnosis, in comparison to other patients (P < 0.05) (Figure 7C). Low or null expression of GM2 synthase entailed for patients a hazard ratio of 3.5 in comparison to high expression (P < 0.01).

Overall, melanomas that showed high expression of both GM1 and GM2 synthases were associated with an overall survival longer than 5 years, while the lack of both these glycosyltransferases increased the hazard ratio to 6.325 (P < 0.01) (Figure 7D). All melanoma cluster 2 cells and the majority of melanoma cluster 3 cells expressed both GM1 and GM2 synthases (Figure 7A).

We also analyzed the expression of other glycosyltransferases involved in ganglioside metabolism such as GD1a synthase, ST3GalVI, ST6GalNAc6 (data not shown) but we did not identify any correlation with patients’ survival.

### Correlation between melanoma cell ganglioside profile and growth/survival and invasive potential

**Figure 8:**
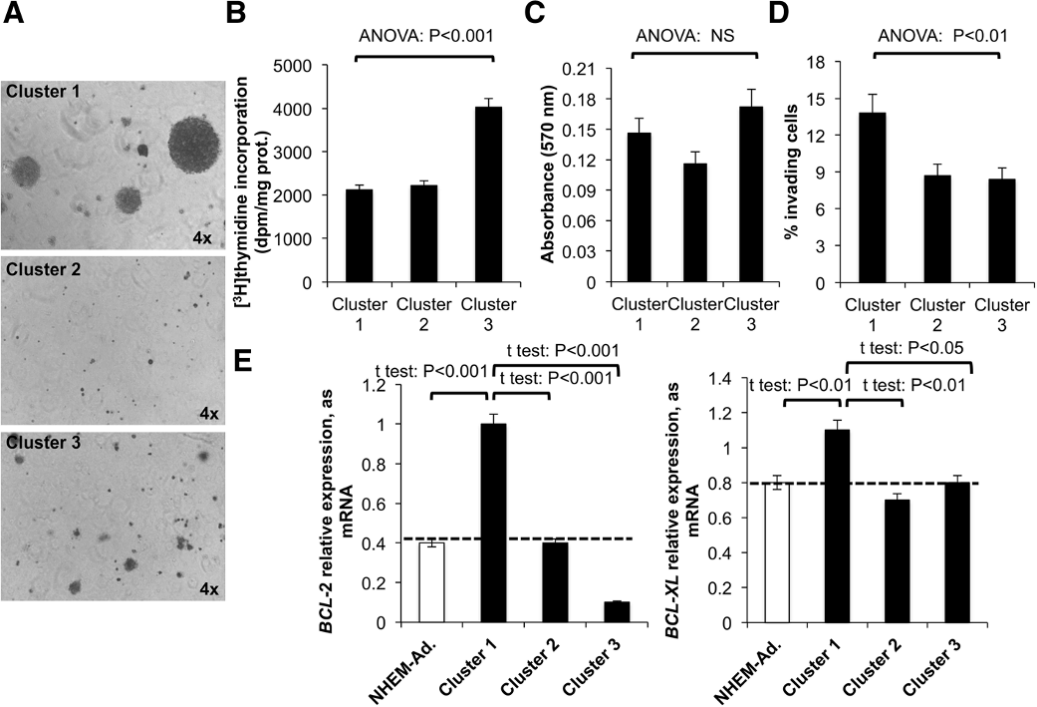
**Growth, invasiveness, and survival potential of melanoma clusters. (A)** Images of colonies formed by representative melanoma cells grouped in cluster 1 (L6), 2 (L1), and 3 (L4) in the soft-agar assay after 3 weeks of incubation at 37°C. Original magnification x4 (Olympus Ix50). Three replicate experiments were performed. Similar results were obtained for other melanoma cell lines according to the clustering. **(B)** Average value of proliferation of melanoma cells grouped in cluster 1, 2, and 3 assessed using [^3^H] thymidine incorporation and **(C)** MTT assay. NS: non significant. Four replicate experiments were performed. **(D)** Average invasiveness potential assessed by Matrigel invasion assay of melanoma cells grouped in cluster 1, 2, and 3. Three replicate experiments were performed. **(E)** Average mRNA expression of *BCL-2* and *BCL-XL* in melanoma cell lines classified in clusters 1, 2, and 3 and in adult melanocytes. Dotted line represents *BCL-2* and *BCL-XL* expression level in adult melanocytes. Values are the mean ± S.D. of four independent experiments.

The anchorage-independent growth potential of melanoma cells was assessed through a soft agar assay. Melanoma 1 cells were able to form more and larger colonies in soft agar than were melanoma cluster 2 cells (4.1-fold; P < 0.01), and than were melanoma cluster 3 cells (2-fold; P < 0.01) (average colony forming efficiency: cluster 1: 53%, cluster 2: 13%; cluster 3: 27%) (Figure 8A). This different potential of growth in soft agar did not reflect a similar inhibition of proliferation capability. In fact, [^3^H] thymidine incorporation was similar between melanoma cluster 1 and 2 cells and even higher in cluster 3 (Figure 8B); MTT assay did not reveal any difference among the three clusters (Figure 8C). Therefore, we could not conclude that the ganglioside profile of the three clusters was determinant for the proliferation rate but only for the tumorigenicity measured as capability to grow in soft agar.

In the Matrigel invasion assay, cluster 1 cells revealed to be more invasive than cluster 2 and 3 cells (+37%; P < 0.01) (Figure 8D).

The resistance to apoptosis evidenced by the expression of *BCL-2* and *BCL-XL* was clearly different between the three clusters: melanoma cells grouped in cluster 1 showed a higher expression of *BCL-2* than melanocytes and also than melanoma cells grouped in clusters 2 and 3 (+150% versus adult melanocytes, P < 0.001; +150% versus cluster 2, P < 0.001; +900% versus cluster 3, P < 0.001) and *BCL-XL* (+38% versus adult melanocytes, P < 0.01; +57% versus cluster 2, P < 0.001; +38% versus cluster 3, P < 0.01) (Figure 8E).

## Discussion

**Figure 9:**
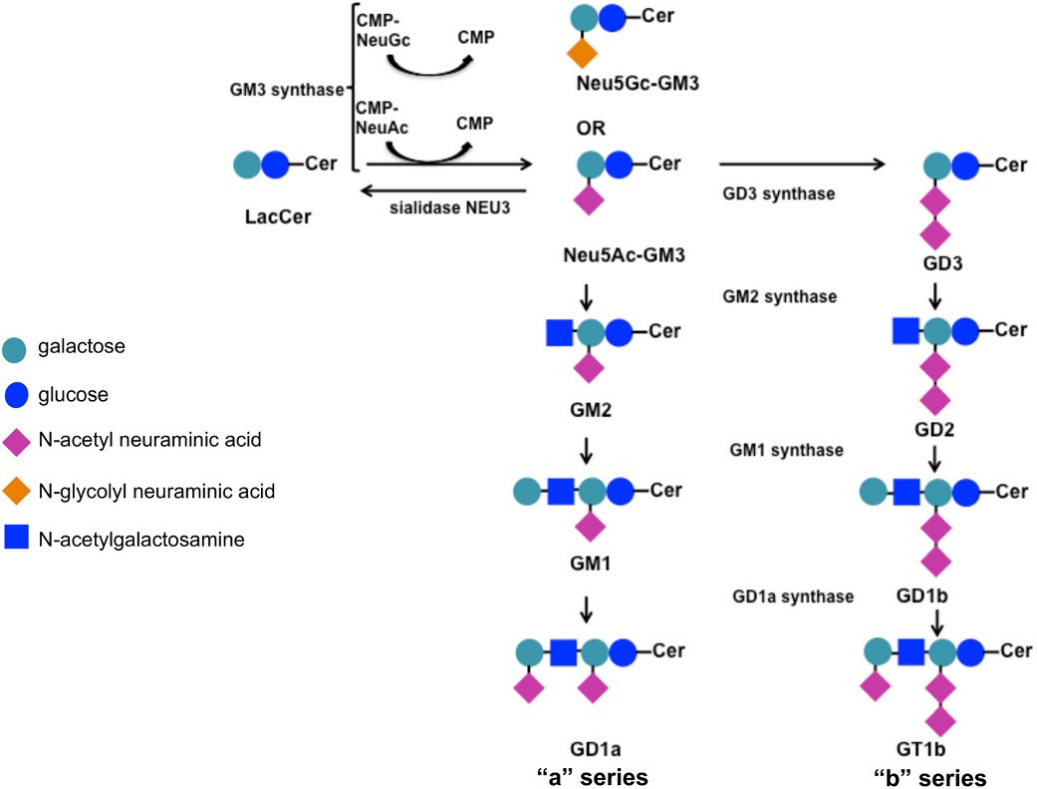
**Schematic picture of ganglioside synthesis pathways.**

The expression of atypical gangliosides deeply marks melanoma [20]. This study demonstrated that melanomas could be classified based on their ganglioside profile and that there is a significant correlation between the ganglioside pattern displayed by melanomas and their biological and clinical behavior. Ganglioside biosynthesis occurs via two major pathways designed “a” (GM2, GM1, GD1a, and GT1a) and “b” (GD3, GD2, GD1b, and GT1b) with a common precursor (GM3) (Figure 9). Intriguingly, melanoma cells that we grouped in cluster 1, retrieved and exacerbated ganglioside biosynthetic pathways typical of neonatal melanocytes, characterized by a high content of GD3 (“b” series) and GM3. GD3 could be considered a typical fetal ganglioside [44]. In melanoma cells, GD3 was demonstrated to enhance the adhesion and proliferation properties by recruiting integrins and signal molecules to lipid rafts [20, 45]. Furthermore, the 9-O acetylation of GD3 (to form 9-OAc-GD3) was described in melanoma [46]: this chemical modification suppresses the pro-apoptotic function of GD3 [47]. Moreover, 9-OAc-GD3 is an important regulatory molecule being involved in signal transduction, regulation of growth, and differentiation [47]. Regarding GM3, it should be noted that, in cluster 1, it was principally in the form of Neu5Gc-GM3 instead of Neu5Ac-GM3. N-glycolylneuraminic acid synthesis has been considered to be absent in normal human tissues due to a partial deletion of the gene coding for CMAH [38, 39]. We demonstrated that melanoma cells characteristically expressed *CMAH* cDNA. Nevertheless, also in melanoma cells, *CMAH* gene carried the 92 bp deletion [40]. Thus, it cannot be hypothesized that the synthesis of N-glycolylneuraminic acid could be rescued in melanoma cells. Otherwise, we determined that Neu5Gc-GM3 amount in melanoma cells depended on the external medium. Therefore, it can be supposed that melanoma cells, in particular those grouped into cluster 1, could incorporated N-glycolylneuraminic acid in a faster way, as described for other tumors [38]. It has been previously showed a correlation between *CMAH* mRNA expression and an increased ability of human stem cells to bind and possibly ingest exogenous N-glycolylneuraminic acid [48]. Therefore, it could be speculated that the appearance of CMAH protein, even though enzymatically inactive, could play a role in the incorporation of external N-glycolylneuraminic acid. The incorporation of N-glycolylneuraminic acid *in vivo* could possibly occur from biological fluids. In fact, the presence of Neu5Gc-GM3 in melanoma surgical specimens was previously largely documented [19, 32, 49]. Moreover, melanoma cluster 1 cells were characterized by the highest levels of expression of GM3 synthase: therefore, also N-glycolylneuraminic acid could be recruited faster into GM3, in these conditions. The increase of Neu5Gc-GM3 was demonstrated to enhance the aggressive behavior of cancer. Mouse B16 melanoma cells loaded with Neu5Gc-GM3 showed more lung metastases and increased tumor burden in subcutaneous implants [50]. Moreover, on the contrary of Neu5Ac-GM3, Neu5Gc-GM3 did not inhibit EGF-induced EGFR phosphorylation in A431 cells [51] and down-modulated CD4 molecule in T lymphocytes [52] and dendritic cell differentiation [53]. To further confirm the involvement of GM3 and Neu5Gc-GM3 in melanoma aggressiveness, we demonstrated that a clone isolated from a single melanoma cell line and endowed with higher adhesive and invasive potential, showed a ganglioside pattern composed only by GM3, mainly in the form of Neu5Gc-GM3. Neu5Gc-GM3 was not efficiently hydrolyzed by plasma membrane sialidase and this explains why melanoma cells could accumulate it despite the high expression of *NEU3*. In our melanoma cells, we could not identify d-GM3 in contrast to Liu et al. who quantified d-GM3 as the major GM3 species present in melanoma [18]. Summing up, melanoma cluster 1 cells showed a ganglioside profile that appeared to confer the highest malignant properties in terms of growth in soft agar medium, *in vitro* invasiveness, and expression of anti-apoptotic proteins. Also, the increase of BCL-2 and BCL-XL could be the direct consequence of an altered control of growth factor receptors such as EGFR, due to the increase of both GD3 [54] and Neu5Gc-GM3 [51] on the plasma membrane.

Melanoma cluster 2 cells completely revolutionized the usual pathways of ganglioside biosynthesis present in melanocytes and acquired the expression of GM1 and GM2 synthases. Their ganglioside pattern was enriched by the presence of complex gangliosides such as GD1a and GT1b, belonging to the “a” pathway, which were not revealed in melanocytes, and to the “b” pathway. It was previously demonstrated that the transfection of GM1 synthase cDNA into the melanoma cell line SK-MEL-37 gave rise to the neo-expression of GD1b, GT1b, and GM1 and, in parallel, reduced cell growth and invasion [55]. In melanoma cluster 2 cells, the expression of GM3 synthase was similar to adult melanocytes and, also, the content of GM3 and Neu5Gc-GM3 was significantly less than melanoma cluster 1 cells. Melanomas classified in cluster 3 represented a middle situation between that displayed by melanoma cluster 1 and melanoma cluster 2 with low Neu5Gc-GM3 levels and GM3 synthase expression and the presence of GD1a. Accordingly to these different ganglioside profiles, also the *in vitro* malignant behavior showed by these two clusters was clearly weakened. Thus, it appears realistic that the synthesis of different gangliosides, altering the recruiting and activation of signal molecules, could reflect on the biological properties of melanoma. Significantly, also the clinical behavior was very different: we traced a correlation between melanoma clusters and patients’ survival. In particular, the synthesis of GM3/GD3 and of “a” complex gangliosides appeared to have a worst and good prognostic value, respectively. Intriguingly, melanomas that retrieved a ganglioside metabolism typical of immature melanocytes (cluster 1), showed the worst outcome (median survival lower than 10 months). It could be supposed that this phenotype corresponds to a higher motile behavior: in fact, neural crest-derived melanoblasts are highly migratory cells [56]. Instead, complex gangliosides, such as GD1a, usually denote more differentiated cells [57]. In the past, pioneering studies have indicated that the GM3: GD3 ratio could be followed for the prognosis and therapeutic management of melanoma [58]. Our results demonstrated that the overall cell ganglioside profile and metabolism could be more important than the presence of a single ganglioside type like GD3 or N-glycolyl GM3 for cell biologic features and, therefore, for prognosis.

Sialidase *NEU3* and GD3 synthase genes were significantly up-regulated in melanomas in comparison to melanocytes, possibly as a direct consequence of the increase expression of the transcriptional factor *Sp1*, but we cannot record any significant correlation with patients’ survival. Sialidase NEU3 could be involved in melanoma malignancy decreasing the levels of Neu5Ac-GM3. It should also be noted that previous papers demonstrated that NEU3 is able to modulate cell signaling also inducing minimal changes in ganglioside profile [21, 59] and that *NEU3* up-regulation could be counterbalanced by other modifications concerning the expression of enzymes involved in ganglioside metabolism, including GM3 synthase, inducing not expected effects on gangliosides [60]. Therefore, the impact of NEU3 up-regulation in melanoma could be complex and should be further investigated in forthcoming works. In summary, the following factors appeared to be determinant for melanoma behavior and could have a significant prognostic value: a) the levels of GM3 and GD3; b) the levels of gangliosides GM1, GD1a, GT1b; c) GM3, GM1, and GM2 synthases expression. Therefore, the determination of ganglioside pattern could significantly improve the prediction of clinical outcome and possibly help the design of the most appropriate therapeutic strategy.

## Conclusions

In addition to be classified on the basis of characteristic genetic mutations, different types of melanomas could also be distinguished on the basis of ganglioside profile. This analysis that could be performed in histochemistry also on surgical specimens, could indicate the aggressiveness of the tumor and, therefore, improve the prediction of the clinical outcome.

## Electronic supplementary material

Additional file 1: **Clinical data and ganglioside composition of melanoma cell lines and melanocytes.** Origin of the cell line: VPG primary tumors (black); metastasis (grey). (DOC 76 KB)

## Abbreviations

AJCC: American Joint Committee on Cancer
d-GM3: de-N-acetyl GM3
Neu5Gc-GM3: N glycolyl GM3
Neu5Ac-GM3: N acetyl GM3
VPG: Vertical growth phase
NHEM-Ad: Adult melanocytes
NHEM-Neo: Neonatal melanocytes
SPG: Sialyl-paragloboside
CMAH: CMP-NeuAc hydroxylase
GM3 synthase: ST3 beta-galactoside-alpha-2,3-sialyltransferase-5
GD3 synthase: ST8 alpha-N-acetyl-neuraminide-alpha-2-8-sialyltransferase
GM2 synthase: Beta-1,4-N-acetyl-galactosaminyltransferase-1
GM1 synthase: UDP-gal,betaGlcNAc-beta-1,3-galactosyltransferase
GD1a synthase: ST6 (alpha-N-acetyl-neuraminyl-2,3-beta-galactosyl-1,3)-N-acetylgalactosaminide alpha-2,6-sialyltransferase.
