# Supplementary material for: Molecular subtyping of metastatic melanoma based on cell ganglioside metabolism profiles
Source: BMC Cancer. 2014 Aug 1;14:560. doi: 10.1186/1471-2407-14-560 (PMC4132924; doi:10.1186/1471-2407-14-560)
Supplement: Supplementary file 1 — Additional file 1: Clinical data and ganglioside composition of melanoma cell lines and melanocytes. Origin of the cell line: VPG primary tumors (black); metastasis (grey). (DOC 76 KB) [file 12885_2014_4756_MOESM1_ESM.doc]

**Tab.1. Clinical data and ganglioside composition of melanoma cell lines and melanocytes. Origin of the cell line: VPG primary tumors (black); metastasis (grey).**

|  |  |  | **% ganglioside** | | | | | | |
| --- | --- | --- | --- | --- | --- | --- | --- | --- | --- |
| **Cell line** | **Origin of the cell line** | **AJCC Stage** | **GT1b** | **GD2** | **GD1a** | **GD3** | **GM1/sialyl paragloboside (SPG)** | **GM2** | **GM3** |
| **L26** |  | IIIb | n.d. | n.d. | 7.1 | 5.2 | 7.2 | 26.6 | 52.9 |
| **L27** |  | IIIc | n.d. | n.d. | 6.1 | 17.2 | 13.7 | 8.7 | 54.3 |
| **L3** |  | IV | n.d. | n.d. | n.d. | 18.8 | 9.2 | 11.6 | 60.4 |
| **L40** |  | IIIb | n.d. | n.d. | n.d. | 24 | 3 | 8 | 64 |
| **L17** |  | IIIc | n.d. | n.d. | 8.4 | 28.4 | 21.1 | n.d. | 42.1 |
| **L33**  **CLUSTER 1** |  | IIb | n.d. | n.d. | n.d. | 31.2 | 10.9 | n.d. | 58 |
| **L5** |  | IIIb | n.d. | n.d. | n.d. | 28.5 | 10.2 | n.d. | 61.3 |
| **L25** |  | IIIc | n.d. | n.d. | n.d. | 29.7 | 7.7 | n.d. | 62.6 |
| **L23** |  | IIIc | n.d. | n.d. | n.d. | 17.6 | 4.7 | n.d. | 77.7 |
| **L22** |  | IIIc | n.d. | n.d. | n.d. | 22.7 | 7.4 | n.d. | 69.9 |
| **L6** |  | IV | n.d. | n.d. | n.d. | 24.6 | 10 | n.d. | 65.5 |
| **L9** |  | IIIb | n.d. | n.d. | n.d. | 25.7 | 9.1 | n.d. | 65.2 |
| **L29**  **CLUSTER 2** |  | IV | n.d. | n.d. | n.d. | 62.4 | 13 | n.d. | 24.7 |
| **L34** |  | IV | 14 | 13.9 | 6.9 | 48.4 | 4.2 | 6.3 | 6.4 |
| **L1** |  | IIIa | 9.4 | 6.3 | 12.8 | 39.1 | 9.4 | 7.6 | 15.3 |
| **L19** |  | IIIa | 2.4 | 9 | 15.5 | 45.3 | 5 | 12.3 | 10.5 |
| **L14** |  | IIc | n.d. | 1.3 | 21.3 | n.d. | 9.4 | 55 | 13.1 |
| **L18**  **CLUSTER 3** |  | IIb | n.d. | 4.8 | 7.4 | 19.4 | 6.4 | 34.4 | 27.5 |
| **L4** |  | IIc | n.d. | 3.4 | 22.9 | n.d. | 17.8 | 20.7 | 35.8 |
| **L38** |  | IIIb | n.d. | 8 | 18 | 13 | 4 | 15 | 40 |
| **L39** |  | IIIb | n.d. | n.d. | n.d. | 30 | 10 | 18 | 40 |
| **L2** |  | IV | 4.4 | 13.3 | 4 | 30 | 6.5 | 14.6 | 27.2 |
| **L16** |  | IIIb | 3.9 | 12.2 | n.d. | 35.1 | 9.8 | 16.9 | 22.2 |
| **NHEM-Ad** | | | n.d. | n.d. | n.d. | 6.2 | 14.1 | n.d. | 79.7 |
| **NHEM-Neo** | | | n.d. | n.d. | n.d. | 22.8 | 7.2 | n.d. | 70 |
